# Aramid Fibers Modulated Polyethylene Separator as Efficient Polysulfide Barrier for High-Performance Lithium-Sulfur Batteries

**DOI:** 10.3390/nano12152513

**Published:** 2022-07-22

**Authors:** Jifeng Gu, Jiaping Zhang, Yun Su, Xu Yu

**Affiliations:** 1 College of Physics and Electronic Engineering, Xinxiang University, Xinxiang 453003, China; zk_gujifeng@163.com (J.G.); wdsysx@163.com (J.Z.); bit-1@163.com (Y.S.); 2 School of Chemistry and Chemical Engineering, Yangzhou University, Yangzhou 250002, China

**Keywords:** nanoporous, aramid fibers, polysulfides, Li–S battery

## Abstract

The separators with high absorbability of polysulfides are essential for improving the electrochemical performance of lithium–sulfur (Li–S) batteries. Herein, the aramid fibers coated polyethylene (AF-PE) films are designed by roller coating, the high polarity of AFs can strongly increase the binding force at AF/PE interfaces to guarantee the good stability of the hybrid film. As confirmed by the microscopic analysis, the AF-PE-6 film with the nanoporous structure exhibits the highest air permeability by the optimal coating content of AFs. The high absorbability of polysulfides for AF-PE-6 film can effectively hinder the migration of polysulfides and alleviate the shuttle effect of the Li–S battery. AF-PE-6 cell shows the specific capacity of 661 mAh g^−1^ at 0.1 C. After 200 charge/discharge cycles, the reversible specific capacity is 542 mAh g^−1^ with the capacitance retention of 82%, implying the excellent stability of AF-PE-6. The enhanced cell performance is attributed to the porous architecture of the aramid layer for trapping the dissolved sulfur-containing species and facilitating the charge transfer, as confirmed by SEM and EDS after 200 cycles. This work provides a facile way to construct the aramid fiber-coated separator for the inhibition of polysulfides in the Li–S battery.

## 1. Introduction

Lithium–sulfur (Li–S) batteries with a high energy density (2600 Wh kg^−1^) and high theoretical specific capacity (1675 mAh g^−1^) have been considered the promising energy storage system in practical applications [1,2]. However, the poor electrical conductivity, large volume expansion, and the shuttle mechanism of polysulfides are still important factors to affect the performance of Li–S batteries, such as the fast degradation of specific capacity [2,3,4,5,6,7]. The shutting mechanism is induced by the free migration of polysulfides anions between cathode and anode during the charge/discharge process, which not only reduces the utilization of active materials but also results in the low columbic efficiency of Li–S batteries [8,9,10].

Recently, the efforts in the modification of host materials have been reviewed to solve the solubility and diffusion of polysulfides [11,12,13]. The separator is an important component to guarantee the safety of Li–S batteries by separating the cathode and anode without direct contact. The modification of separators is an effective strategy to solve the polysulfide diffusion in an organic electrolyte, such as coating some porous materials on the separator surface, which can act as a physical barrier to block the polysulfide diffusion during cycling. The coating of porous carbon or carbon nanotubes on commercial polypropylene (PP) separators has been reported to show the improved electrochemical performance for Li–S batteries by effectively trapping the polysulfides [14,15]. Meanwhile, many reported lectures demonstrate that the high porosity of metal oxides or sulfides (MnO_2_, TiO_2_, Al_2_O_3_, and MoS_2_) as a coating layer can also enhance the capacitive behavior of Li–S batteries due to their polarity, hydrophilic property, and high absorbability by interacting with polysulfides [15,16,17,18,19,20]. However, these porous materials are almost nonpolar or weakly polar conductive materials, which only own a single physical barrier to the dissolution of lithium polysulfides. Considering the high polarity surface of the separator, the coating layer may be peeled off from the separator after long-term charge/discharge cycles because of the weak binding force between inorganic coating materials and the separator.

The exploration of high polarity coating materials is necessary to be studied. Aramid fibers (AFs) serving as new building blocks have attracted attention owing to the low cost, high strength, high-temperature resistance, and excellent dimensional stability [21,22,23], which is favorable to improve the mechanical property of the separator and the ionic conductivity of batteries [22]. Yang and co-workers report an aramid nanofiber/bacterial cellulose (ANFs/BC) composite, which exhibits increased ionic conductivity and interfacial compatibility. The electrochemical performance is significantly improved as the optimal ANFs/BC as the separator in the battery cell [22]. However, the aramid fibers coating on polyethylene (PE) membrane with the nanoporous structures as the separator for Li–S batteries has yet to be explored.

Herein, the aramid fibers coating on PE membrane (AF-PE) was prepared, and the effect of coating content on the porosity of AF-PE films is discussed. AF-PE-6 with the optimal coating content shows better air permeability than other AF-PE films. As the AF-PE-6 film applies as the separator in a lithium–sulfur battery, the electrochemical performance is dramatically enhanced in contrast with pristine PE films, such as the high specific capacity of 661 mAh g^−1^ at 0.1 C, and 38.9% of capacity retention from 0.1 to 1 C. Especially, the specific capacity remained the value of 542 mAh g^−1^ after 200 charge/discharge cycles. This work provides a facile synthetic route to prepare the high polarity separators to further enhance the capacitive performance of the Li–S batter.

## 2. Experimental Section

### 2.1. Materials

The PE microporous separators (GRE-20, Green Inc, Xinxiang, China, 20 μm) were used as base membranes. The organic electrolyte was made by dissolving 1M bis-(trifluoromethane) sulfonamide lithium (LiTFSI) into a 10 mL mixed solvent of dimethoxyethane (DME) and 1,3-dioxolane (DOL) in 2:1 volume ratio to test the electrochemical performances. Aramid fibers were purchased from Dongbang special fiber Co., Ltd. (Zhangjiagang, China).

### 2.2. Preparation of AF-PE Separators

Aramid fibers have a limited solubility in NMP (or DMF, DMAc); however, fast dissolution could be obtained after adding a certain amount of salt (LiCl or CaCl_2_). Hence 3 g of aramid fibers and 0.3 g of LiCl (Aladdin Industrial Co., Shanghai, China) were mixed with the mass ratio of 10:1 as the co-solvent and subsequently immersed in 50 g of N-Methy pyrrolidone (NMP, Aladdin Industrial Co., Shanghai, China) under magnetic stirring at 65 °C for 8 h. The obtained transparent solution was coated on polyethylene (PE) separators by a simple roller coating technology, and then the separator was immersed in deionized water for about 5 min. Finally, the separator was dried at 55 °C for 12 h under vacuum conditions and named AF-PE-6. As for comparisons, the different mass ratios of aramid fibers and co-solvent (5:1 and 15:1) were treated at the same synthetic process and noted as AF-PE-3 and AF-PE-9.

### 2.3. Characterization

Surface morphology of AF-PE and PE films with different strains were observed using Zeiss Scanning electron microscopy (SEM, SIGMA, ZEISS, Oberkochen, Germany) and energy dispersive X-ray analysis (EDX) at an accelerating voltage of 5 kV. The average pore size, pore size distribution and porosity were evaluated using a through pore size analyzer instrument (porosimeter 3G, Quantachrome Instruments, Boynton, FL, USA). Permeability test was evaluated using Gurley test instruments 4410N.

### 2.4. Electrochemical Measurements

The electrochemical performance of AF-PE separators was measured by assembling CR2032 type coin cells in the glove box (Mbraun, M. Braun Inertgas-Systeme GmbH, Garching, Germany) at Argon atmosphere. The sulfur cathodes were prepared by a conventional slurry coating method with a doctor blade, and the detailed process was listed as follows: 80 wt% of pure sulfur (Sigma-Aldrich, St. Louis, MO, USA), 10 wt% of carbon black (Super P), and 10 wt% of PVDF were placed in an agate mortar and ground with adding few drops NMP as the solvent for 40 min. The obtained slurry was pasted onto the aluminum foil and dried in a vacuum oven at 60 °C overnight. The mass loading of the sulfur cathodes was about 2.5 mg cm^−2^. The sulfur cathode acted as the working electrode, pristine PE and AF-PE-6 films as the separator and lithium metals (Sigma-Aldrich) as the anode electrode, the assembled batteries were named pristine PE cell and AF-PE-6 cell. Galvanostatic charge/discharge (GCD) measurement was performed at different current densities in the voltage range of 1.8–2.8 V with program-controlled battery test equipment (LAND CT2001A, Wuhan LAND Electronic Co.Ltd., Wuhan, China). Ionic conductivities of the membrane with electrolyte were measured by sandwiching it between two stainless steel electrodes, and the ionic conductivity was calculated using formula: σ = d/RA, where d was the thickness, A was the separator effective area of a membrane and R was the bulk resistance. Ionic conductivities and the electrochemical impedance spectroscopy (EIS) were measured by an electrochemical workstation (CHI660E, Shanghai, China) over a frequency range of 1 Hz–100 kHz with an AC voltage amplitude of 5 mV. For comparison, the assembled CR2032 coin cell with commercial PE film as a separator was measured under the same condition.

## 3. Results and Discussion

**Figure 1:**
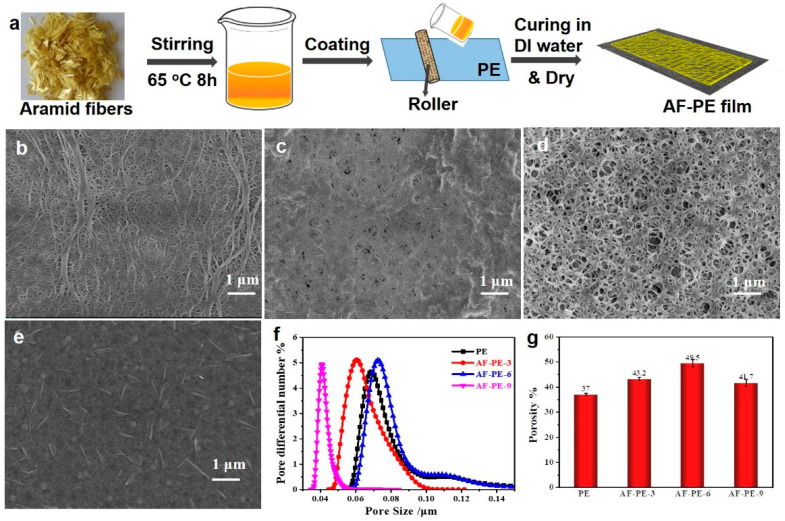
(**a**) Schematic illustration of the preparation of AF-PE films. SEM images of: (**b**) pristine PE film; (**c**) AF-PE-3; (**d**) AF-PE-6; and (**e**) AF-PE-9; (**f**) The pore size distribution of pristine PE, AF-PE-3, AF-PE-6 and AF-PE-9 films; (**g**) Porosity of PE and aramid-coated separator.

The aramid fibers coating on polyethylene (PE) membrane (AF-PE) were prepared by combining a simple bar coating process and low-temperature vacuum drying methods in Figure 1a. The existence of a co-solvent of aramid/LiCl is favorable to obtain the homogeneous solution, and the treatment in DI water aims to cure the film and remove the residues or co-solvent. Scanning electron microscopy (SEM) was characterized to reveal the morphological structure of AF-PE and pristine PE films. It can be found that the pristine PE film exhibits cross-linked internetworks and porous structures in Figure 1b. The morphological structure of AF-PE films is affected by the coating content of aramid fibers on the PE surface by filling with the porous structure (Figure 1c–e). AF-PE-6 with optimal coating content of AF shows the increased porosity including mesopores and micro-pores in Figure 1d, which is better than AF-PE-3 with the insufficient content of AFs and AF-PE-9 with the over-coating of AFs. The average pore size of all samples is shown in Figure 1f, and the AF-PE-6 owns the value of 98.9 nm, which is larger than that of AF-PE-3 (81.1 nm), AF-PE-9 (59.9 nm), and is smaller than pristine PE film (104.7 nm), respectively. The porous structure is favorable for the fast electrolyte diffusion, while the tortuous pores of AF-PE-6 can localize the polysulfide species diffusing from the cathode to the anode sites. The porous structure of the aramid-coated separator can also be quantitatively characterized by measuring the Gurley value and porosity. The air permeability of AF-PE is affected by the coating content of AF. In comparison to pristine PE film (278 s), the Gurley value of AF-PE-3 increased resulting from the AF coating. However, the Gurley value of AF-PE-6 (440 s) is smaller than these of AF-PE-3 (571 s) and AF-PE-9 (760 s), implying a better air permeability of AF-PE-6. The porosity of AF-PE-6 (49.5 + 1.5%) is higher than pristine PE (37 + 0.5%), AF-PE-3 (43.2 + 0.7%) and AF-PE-9 (41.7 + 1.4%), which is attributing to the optimal coating content of AFs in Figure 1g. This result is attributed to the optimal coating content of AF because the insufficient content of AF blocks the original pores of pristine PE film and the overloading of AFs results in the increased densification of AF-PE-9 film.

**Figure 2:**
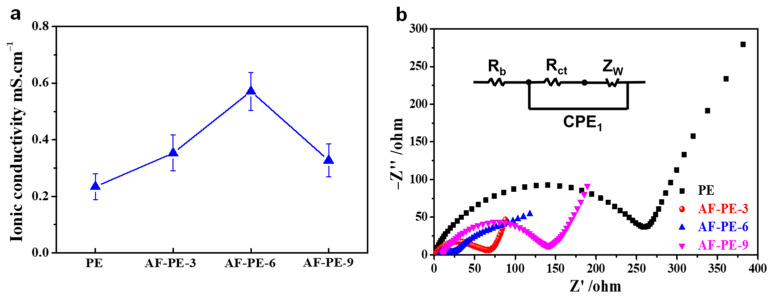
(**a**) The ionic conductivity of PE and AP-PE films, (**b**) Nyquist plots of pristine PE, AF-PE-3, AF-PE-6 and AF-PE-9.

To initially evaluate the quality of AF-PE and pristine PE film, the ionic conductivity of separators and the electrochemical performance of the constructed Li–S cells are compared with the AF-PE films and commercial sulfur as the separators and cathode material. As shown in Figure 2a, the ion conductivity value of the AF-PE-6 film can reach up to about 0.57 mS cm^−1^, which is almost 2.5, 1.6 and 1.7 times larger than that of PE (0.23 mS cm^−1^), AF-PE-3 (0.36 mS cm^−1^) and PF-PE-9 (0.33 mS cm^−1^). The large ion conductivity for AF-PE-6 is assigned to the increased porosity. The resistance of the fresh Li–S cells is confirmed by electrochemical impedance spectroscopy (EIS) in Figure 2b. The semicircle at the high-to-medium frequency and an inclined line at low frequency correspond to the charge transfer resistance (*R_ct_*) and mass transfer process, respectively. AF-PE-6 cell shows a smaller diameter of the semicircle and steeper slope line than that of pristine PE cell, implying a faster charge transfer kinetics. The *R_ct_* value for AF-PE-6 cell (21.56 Ω) is smaller than that of PE cell (260.1 Ω), attributing to the enhanced affinity and wettability for the accumulation of polar liquid electrolytes by coating optimal content aramid fibers [24,25]. Especially, AF-PE-9 cell shows the highest *R_ct_* value, and the overloading of aramid fiber can increase the density of film and decrease the average pore size, which is not favorable for the electrolyte ion passing through the film.

**Figure 3:**
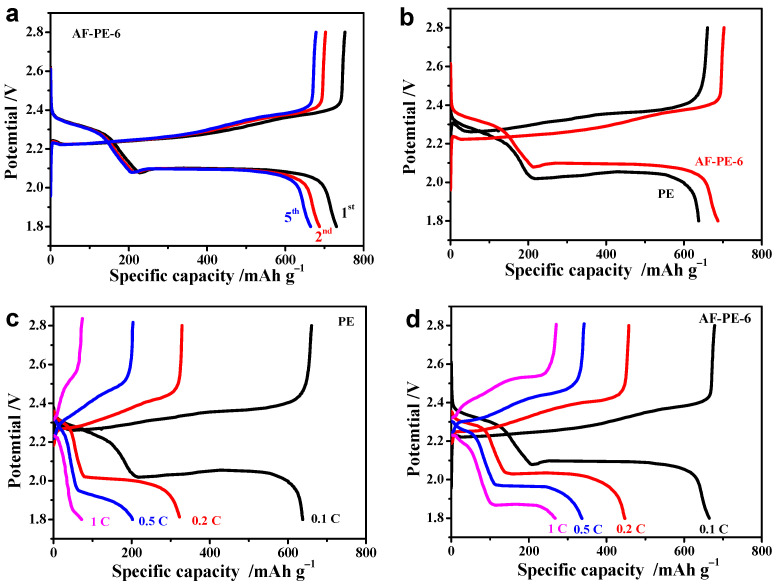
(**a**) The 1st, 2nd and 5th cycle of GCD curves of AF-PE-6 cell; (**b**) The GCD curves of pristine PE and AF-PE-6 cells. The GCD curves at different C rates of (**c**) pristine PE; and (**d**) AF-PE-6 cells.

The electrochemical performance of pristine PE cell and AF-PE-6 cell is further confirmed by galvanostatic charge/discharge (GCD) at 0.1 C with the applied potential range from 1.8 to 2.8 V. Figure 3b show the GCD curves of pristine PE cell and AF-PE-6 cell, and both cells display two voltage plateaus arising from the two steps redox reaction of elemental sulfur with metallic lithium during the discharge process. Interestingly, AF-PE-6 cell owns a lower charge plateau potential and higher discharge plateau potential than this pristine PE cell, and a smaller potential separation between charge and discharge plateau indicates a better kinetic behavior for AF-PE-6 cell. For the first cycle, the discharge capacity of AF-PE-6 cell is 731 mAh g^−1^ with the charge capacity of 753 mAh g^−1^ at 0.1 C, which decreases to 687 mAh g^−1^ for the second cycle and 665 mAh g^−1^ for the fifth cycle, respectively. The degradation of the discharge capacity can be attributed to the formation of the SEI layer. It can be found that the discharge capacity is almost stable after 5 charge/discharge cycles. The GCD curves of pristine PE cell and AF-PE-6 cell at 0.1 C are shown in Figure 3b, and the discharge capacity of AF-PE-6 cell is 687 mAh g^−1^ higher than that of pristine PE cell (638 mAh g^−1^). As the C rate increased by a factor of 10 (Figure 3c), pristine PE cell owns the discharge capacity of 72 mAh g^−1^ with the charge capacity of 74 mAh g^−1^, and the calculated capacity retention is only 11.2%, respectively. The discharge capacity is 267 mAh g^−1^ for AF-PE-6 cell with a capacity retention of 38.9%, which is higher than that of pristine PE cell (Figure 3d). The high discharge capacity and good capacitance retention for AF-PE-6 cell reflect the enhanced sulfur utilization to tolerate the high charge currents.

**Figure 4:**
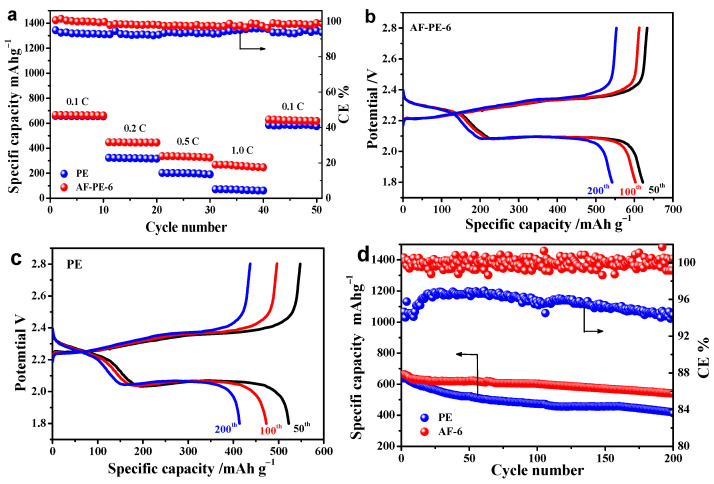
(**a**) The specific capacity and the related coulombic efficiency of PE and AF-PE-6 cells at various C rates with 10 cycles for each. The GCD curves of: (**b**) AF-PE-6 and (**c**) PE cells at the 50th, 100th and 200th cycles; (**d**) The cyclic performance of PE and AF-PE-6 cells and the related coulombic efficiency.

Furthermore, the stability of pristine PE and AF-PE-6 cells were evaluated at different C rates with 10 cycles for each in Figure 4a. As the C rates increased from 0.1 C to 1 C, the specific discharge capacity is decreased from 661 to 247 mAh g^−1^ for AF-PE-6 cell, and then the discharge capacity keeps at the value of 618 mAh g^−1^ as the C rate returns to 0.1 C. The loss of discharge capacity is only 43 mAh g^−1^. In comparison, the discharge capacity of pristine PE cell dramatically decreases from 656 mAh g^−1^ at 0.1 C to 61 mAh g^−1^ at 1 C. The improved electrochemical performance of AF-PE-6 cell can be attributed to the limitation of polysulfides at the sulfur cathode/aramid-coated separator interface by physical absorption and electrochemical deposition. The cycling test of pristine PE and AF-PE-6 cells is carried out by GCD at 0.1 C for 200 cycles. Figure 4b,c shows the GCD curves of pristine PE and AF-PE-6 cells at different cycles. It can be found that the specific discharge capacity of AF-PE-6 cell is only 622, 603 and 542 mAh g^−1^ loss for the 50th cycle, 100th and 200th cycles. After 200 charge/discharge cycles, the capacity retention of AF-PE-6 cell (81.9%) is higher than that of the pristine PE cell (64.8%) in Figure 4d. Meanwhile, the related coulombic efficiency of AF-PE-6 cell is higher than that of the PE cell. The excellent rate capability and cyclic stability for AF-PE-6 cell can be attributed to the blocking effect of aramid coating on the polysulfides.

**Figure 5:**
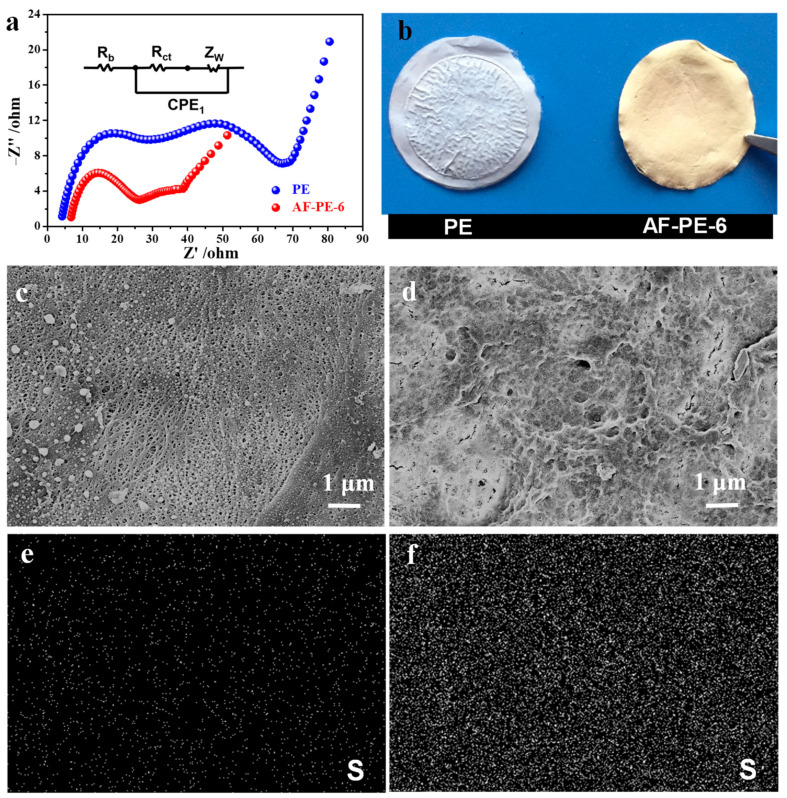
(**a**) Nyquist plots and (**b**) the optical images of pristine PE film and AF-PE-6 film after 200 cycles. SEM images of (**c**) pristine PE and (**d**) AF-PE-6 films after 200 cycles. Elemental mapping of S of (**e**) pristine PE and (**f**) AF-PE-6 films after 200 cycles.

Figure 5a shows the EIS result of pristine PE and AF-PE-6 cells after 200 GCD cycles. AF-PE-6 cell exhibits two depressed semicircles at high and middle frequency, and an inclined line at low frequency. The R_s_ value of AF-PE-6 cell is smaller than that of pristine PE cell, implying the efficient inhibition of polysulfides by coating aramid fibers. In comparison to pristine PE cell, the *R_ct_* values of AF-PE-6 cell is significantly decreased after 200 cycles, and a significant decrease in charge transfer resistance is attributed to the dissolution and redistribution of the active materials during the chemical activation process [26].

The change in surface morphology of pristine PE and AF-PE-6 films after 200 cycles is probed by SEM. In comparison to pristine PE film, the color of AF-PE-6 film becomes yellow (Figure 5b), arising from the interception and adsorption of soluble polysulfides by the optimal coating content of aramid fibers. Figure 5c,d are the SEM images of pristine PE and AF-PE-6 films after the cycling test. In comparison to pristine PE film, AF-PE-6 film with a small pore size can not only block the polysulfides, but also as a barrier for trapping the polysulfides. The elemental mapping of S for pristine PE and AF-PE-6 films is disclosed in Figure 5e,f. The existence and uniform distribution of S can be found on the surface of films. However, the content of elemental S on AF-PE-6 film is more than that of PE film, indicating that the coating of aramid fiber is favorable for the absorption of polysulfides. Therefore, this work provides a promising strategy to construct the separators to efficiently suppress the shuttling mechanism of polysulfides for Li–S batteries.

## 4. Conclusions

The AF-PE-6 film with high porosity has been prepared and acts as the separator for the Li–S battery. The morphological structure of AF-PE-6 is characterized by SEM, and the effect of coating content of AFs on the porosity of hybrid films is discussed. The air permeability of AF-PE-6 is superior to other control samples determined by the optimal coating content of AFs, which showed the enhanced electrochemical performance of the Li–S battery. The specific discharge capacity is 661 mAh g^−1^ at 0.1 C, and 247 mAh g^−1^ of specific capacity is maintained at the C rate increased by a factor of 10, which is better than pristine PE cell. The high specific capacity and good rate capability of AF-PE-6 cell are attributed to the high porosity of the separator and the increased absorbability of polysulfides by coating AFs.

## Data Availability

The data that support the findings of this study are available from the corresponding authors upon reasonable request.
